# Socioeconomic impact of high-cost drugs in Brazilian dermatology. Legal and financial aspects, and impact on clinical practice^☆^

**DOI:** 10.1016/j.abd.2020.08.010

**Published:** 2021-01-30

**Authors:** Renan Tironi Giglio de Oliveira, Giovana Larissa Prado Leite Agostinho, Rubens Granja, Luiza Keiko M. Oyafuso, Paulo Ricardo Criado

**Affiliations:** a Dermatology Residence Program, Centro Universitário FMABC, Santo André, SP, Brazil; b Dermatology Course, Centro Universitário FMABC, Santo André, SP, Brazil; c Civil Law Discipline, School of Law, Universidade de São Paulo, São Paulo, SP, Brazil

**Keywords:** Dermatology, Legal health issues, Specialized pharmaceuticals

## Abstract

The technological advancement of the pharmaceutical industry, resulting from the techniques of molecular biology and expansion of the knowledge of immunopathogenesis, has modified the therapeutic arsenal used in dermatology. Scientific research and regulatory standards cause drug costs to rise, thus making their use impossible in most public policies. In order to make high-cost drugs viable in the public health network, the Specialized Pharmaceutical Assistance Component was created. However, despite the frequent incorporation of medications, the constant requirement of drugs through lawsuits leads to exorbitant costs to the state budget. The present work analyzed through a descriptive observational study, the current situation of the Specialized Component and the involvement of medicines used in dermatology, through legal reviews, financial analyses, and medical articles, aiming at future incorporations for the specialty. When assessing the legal demands for dermatological drugs in the state of São Paulo, the specialty still has a low participation and psoriasis is the main disease involved in requiring drugs through the judicial system in the state. New methods of access to raw materials must be created to reduce legal issues. Cost-effectiveness and public planning studies are mandatory for incorporating new dermatological therapies.

## Introduction

The Brazilian Federal Constitution broadly states that health is a duty of the state and a fundamental right of all citizens, and that right should be guaranteed by comprehensive therapeutic and pharmaceutical assistance, instituted by law 8.080/1990, with the creation of the Unified Health System (Sistema Único de Saúde [SUS]).^1^, ^2^ The Federal Government, through the Ministry of Health (MH), is responsible for coordinating the National Pharmaceutical Assistance Policy (Política Nacional de Assistência Farmacêutica [PNAF]), whose objective is to promote the rational use of medicines.^3^

Pharmaceutical assistance is the set of actions and measures, approved by the PNAF, aimed at the promotion, protection, and recovery of health, based on the principles of universality, integrality, and equity. After the implementation of Ordinances No. 2,577/2006 and 204/2007, pharmaceutical assistance was organized into Components. The Basic Component aims to provide medicines for the most prevalent diseases in the population (such as diabetes and systemic arterial hypertension) and is financed with resources from three sources (Federation, States, and Municipalities). In turn, the Strategic Component aims to provide medicines for the control of endemic diseases, under the exclusive financial responsibility of the MH.^1^, ^3^

Since 2010, the MH has also provided the Specialized Pharmaceutical Assistance Component (Componente Especializado de Assistência Farmacêutica [CEAF]). For inclusion in CEAF, drugs must be approved by the National Commission for the Incorporation of Technologies (Comissão Nacional de Incorporação de Tecnologias [CONITEC]) and integrated into the Clinical Protocols and Therapeutic Guidelines (Protocolos Clínicos e Diretrizes Terapêuticas [PCDT]).^2^, ^3^

With the rapid technological expansion, several highly effective treatments appear in the pharmaceutical market and are constantly associated with high financial investments. For budgetary reasons, the speed of inclusion of certain technologies in CEAF does not keep pace with the speed of the pharmaceutical industry, which caused the emergence of the phenomenon of lawsuits to obtain medicines, increasing public spending in the healthcare area.^1^, ^3^

In dermatology, multiple clinical outcomes are dependent on high-cost therapies, in addition to specific investments by the responsible agencies, whether public or private.

## Methods

The main objective of this descriptive observational study was to demonstrate – through case series assessments, legal considerations (decisions in medical law, articles of the federal constitution) and financial analyses (national and international pharmaceutical-economic studies and management analyses in public healthcare) – the importance of the cautious use of medicines of the specialized component in Brazilian dermatology. It was structured primarily around the assessment of legal aspects, financial aspects, and monitoring of the technological perspectives of dermatology in the coming years. It is targeted at dermatologists, public policy managers, and national law and healthcare professionals responsible for drug access programs.

## Legal aspects

### History of laws and legal protections

According to articles 6, 194, 196, 198, and 200 of the Constitution, the State must bear the responsibility to offer health as a supreme, basic, social, and fundamental right to any and all Brazilian citizens. In this way, the following principles are ensured: equality in the care of patients, respect for the principle of human dignity, and the “minimum existential”.^4^, ^5^, ^6^, ^7^, ^8^

In 1990, two important laws were enacted: Organic Law (Lei Orgânica [LO]) 8.080/90, which established the basis for the operation of SUS, and Law 8,142/90, which determines the creation of Health Conferences and Councils. Article 6 of the LO determines the implementation of comprehensive therapeutic assistance actions (including pharmaceutical) and the assessment of the impact that technologies cause to health.^5^ In 2006, with the formulation of the Health Pact (Ordinance GM/MS No. 399) and in 2011 by Decree 7.508/11, certain gaps in the SUS strategic process were filled, setting forth national policies and guidelines.^9^

The legal provisions aimed at pharmaceutical assistance were first implemented by Ordinance No. 3,916/98, establishing the elaboration of the National Medicines Policy and the creation of the National List of Essential Medicines (Relação Nacional de Medicamentos Essenciais [RENAME]).^10^ CEAF was intended for the exceptional dispensation of medicines for the treatment of chronic, rare, or low prevalence diseases, with an indication of the use of medicines of high cost per unit or with high annual added cost. These drugs are financed by the MH and state health departments, and are included in the component list after the PCDT for each disease is created.^11^

Law 12,401/2011 defined the criteria and deadlines for the incorporation of new technologies in the public health system, requiring prior registration of the product at the National Health Surveillance Agency (Agência Nacional de Vigilância Sanitária [ANVISA]) and also established that the MH, assisted by CONITEC, has the attributions for incorporating, excluding, or altering the approved list of drugs and products.^12^

### The phenomenon of Judicialization in healthcare policies

The Judiciary Power (Poder Judiciário [PJ]) has the mission to apply justice through the employment of the constitution's laws and regulations. Unlike the other powers, the PJ is not elected by the society in a democratic process and its representatives exercise their functions for their life period.^7^, ^13^

In the field of healthcare policies, the PJ has had an increasing role in accomplishing the right to health. The so-called phenomenon of Judicialization has been translated as a guarantee of access to goods and services through actions from the PJ aiming to impose certain demands on the Executive Power.^13^, ^14^, ^15^

The Brazilian legal system provides two types of processes to guarantee the judicial protection of rights: the individual process (regulated by the Civil Procedure Code) and the collective process (regulated by the Public Civil Action Law and the Consumer Defense Code).^10^

The judicial determinations of benefits and allocations of public resources often disregard the fact that the State operates within well-defined financial, administrative, and institutional parameters, subject to concrete limits, and does not take into account the country's continental dimensions and its heterogenous geography and population.^6^ National law operators sometimes demonstrate little knowledge about public policies in general (the impropriety principle of PJ), especially regarding the technical details of pharmaceutical assistance within SUS, a fact observed in certain court decisions in favor of providing financially unviable drugs for collective distribution.^14^

Between 2008 and 2017, the number of health-related lawsuits increased by 130%, while the total number of lawsuits increased by 50%. According to the MH, in seven years there was an increase of approximately 13 times in its expenses associated with legal claims, reaching R$ 1.6 billion in 2016.^16^ In 2004, the Federal Court of Accounts (Tribunal de Contas da União [TCU]) conducted an operational audit on the financial assistance for the purchase and distribution of exceptional medicines, and found that the legal claims for health assistance caused the emergency reallocation of program resources and discontinued the treatment of regular patients.^11^ Certain reasons are listed to justify legal claims for health assistance in the national scenario, such as those listed in Table 1.^14^

**Table 1:** List of scenarios that determine legal claims for medicines or supplies in health.

Absence of a cohesive discourse on public health policies, generating difficulty in understanding their meaning by the Judiciary, and often, financial burden to the public entity not responsible for pharmaceutical assistance.
Lack of articulation between healthcare managers and the population, so that the uncertainties generated and the absence of communication channels cause dissatisfaction. Patients are often unaware of where and how they can purchase medications.
Distortion of the definitions of constitutional principles by the judges.
Lack of delimitation of the concept of integrality: judges grant medicines based on the theory of immediate, complete, and rightful effectiveness of constitutional norms.
Ignorance of the official lists by doctors and lawyers, resulting in unnecessary lawsuits.
Medical professionals lack of social commitment when prescribing a certain drug without first assessing protocols and public viability
Growth of judicial populism: with pro-patient decisions, new requests arise.
Carelessness with the patient's itinerary: exaggerated bureaucracy associated with disregard in the conservation and shortage of products in reference centers.

### Socioeconomic profile of users

The objective of equity is to guarantee all people equal conditions and equal access to actions and services at different levels of complexity in the health system.

In view of the fact that in some geographical areas, the majority of lawsuits are conducted by private lawyers, the legal claims for health assistance process would collaborate to increase inequities in healthcare access.^9^ Individuals from the most favored sectors of society obtain the right to access certain treatments due to the fact that they are able to receive quality legal defense, making a plural right restricted to an elite.

Another factor that helps delimit the profile of the patient who requires medication through the courts is the greater number of individual actions when compared with collective actions. This reinforces the idea that micro-litigation or micro-justice is a reality that creates challenges for the judiciary and for health management itself.^16^, ^17^, ^18^

In geographical terms, in the most economically active states of the country (such as São Paulo and Rio de Janeiro), there has been a decrease in the success of lawsuits or a reduction in the absolute number of lawsuits, which can mean a greater maturity of these judicial systems and/or better assistance from healthcare systems.^16^ In turn, this efficiency generates a constant interstate migration of patients to receive drug therapy, generating an imbalance in state spending. This phenomenon is commonly observed in the borders between the states of São Paulo, Minas Gerais, Bahia, and Rio de Janeiro.^19^

### Positive aspects of legal claims for health assistance

In general, judicial decisions put pressure on the Executive to emerge from its inertia and inefficiency, as well as make it possible to reverse omissions, illegalities, or abuses.^14^

The National Council of Justice (Conselho Nacional de Justiça [CNJ]) issued Recommendation No. 31/2010 and later Resolution No. 107, establishing the National Health Forum and the State Executive Health Committees in order to monitor and solve healthcare demands. The Supreme Federal Court (Supremo Tribunal Federal [STF]) called a public hearing on healthcare and held a wide discussion on the cost of medicines and treatments not covered by existing public policies and on the responsibilities of the states and municipalities regarding the right to health.^13^, ^17^

The purpose of the Technical Assistance Centers (Núcleos de Assessoria Técnica [NAT]) is to assist magistrates in the judgment of health-related demands, providing technical knowledge for safer decisions.^13^, ^16^, ^17^ As another example of activities carried out by the PJ, the Right to Health Days (Jornadas de Direito a Saude) were created, focused on identifying parameters that could serve as guidelines for the decisions of judges.^7^, ^13^

In practical financial terms, legal claims for health assistance have a direct impact on product pricing, influencing public procurement management. In this context, new criteria for analysis of incorporation and regulation of the price of medicines have emerged. Some are used by the Medicines Market Regulation Chamber (Câmara de Regulação do Mercado de Medicamentos [CMED]), such as production costs, therapeutic utility, economic evaluation, international comparison, and price of other treatments available on the market.^20^, ^21^

In the field of supplementary health, much is discussed about the rights of consumers of health insurance plans and the responsibility of operators in providing the service. Faced with a majority of decisions favorable to users, insurance companies have started to bear the burden of non-covered procedures, even beyond the limits defined by the National Supplementary Health Agency (Agência Nacional de Saúde Suplementar [ANS]). It is noteworthy that certain deferrals in favor of users do not start from a legal basis and are based purely on the articles of the CDC. In turn, possible rejections would probably be absorbed by the SUS, reflecting the economic imbalance of health-related public accounts.^22^, ^23^

### Analysis of the legal claims for dermatological assistance in the State of São Paulo and the current national situation of the specialty

The lack of standardization in the computerized system for monitoring legal actions prevents the establishment of parameters that are reliable for parity, generating bias in the interpretation and erroneous assessments of the indicators.^16^

Data collected from the São Paulo State Department of Health (Secretaria de Estado da Saúde de São Paulo [SES-SP]) in 2018 and delivered in May 2019 to the TCE demonstrate that the legal claims for health procedures accounted for a total cost of R$ 664.7 million.^24^ In studies carried out in 2015, the state of São Paulo was the first in absolute number of lawsuits, but second to the state of Rio Grande do Sul in number of lawsuits per 100,000 inhabitants. Judicial spending at SES-SP decreased by 18.82% between 2016 and 2017, and has since maintained the pattern of reduction over the years.^25^, ^26^

**Figure 1:**
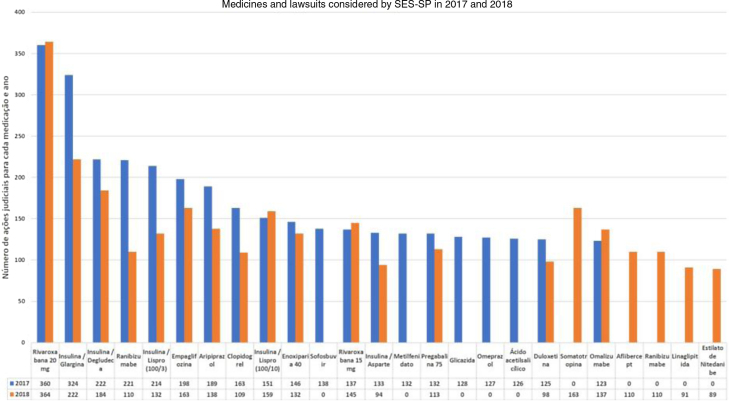
Drugs supplied by SES-SP in the 2017–2018 biennium in compliance with the demands through lawsuits.

In the case of dermatology, there are few studies involving the specialty in the national legal claims for health assistance process. Through the Access Law (Federal Law 12.527/2011), the profile of the dermatological lawsuits at SES-SP in the years 2017 and 2018, assessed by the S-Codes program, was provided. The data indicate a small share of non-biological dermatological drugs, and that the drug omalizumab had a significant role in the total number of judicial requests in that state (123 in 2017 and 137 in 2018, without ICD specification), being the ninth highest in 2018 at SES-SP (Fig. 1).^27^, ^28^

It is important to highlight the permanence of lawsuits for medications that are included in the component lists, such as hydroxyzine solution, ketoconazole cream, and cyclosporine capsules, which reflects the difficulty of access by the population. In terms of the distribution among the municipalities, with the exception of the city of São Paulo, no other regional predominance is observed and there was a greater distribution of lawsuits among the municipalities in 2018.^28^

When comparing with the study carried out in 2006 with data obtained from SES-SP, dermatology was responsible for 4.09% of all lawsuits, and immunobiologicals for psoriasis were a considerable portion of this percentage.^18^

In October 2012, CONITEC published a recommendation against the incorporation of biological drugs (infliximab, etanercept, adalimumab, and ustekinumab) for the treatment of moderate to severe psoriasis in adults. Only at its 66^th^ regular meeting, in 2018, did it recommend the incorporation of adalimumab as the first line of biological treatment after failure of standard therapy and secukinumab as the second line of treatment for moderate to severe psoriasis.^12^ The inclusion of etanercept for pediatric patients and ustekinumab in cases of failure of second line treatment was authorized in the records of the 70^th^ CONITEC Meeting; the use of infliximab remained denied.^29^

**Figure 2:**
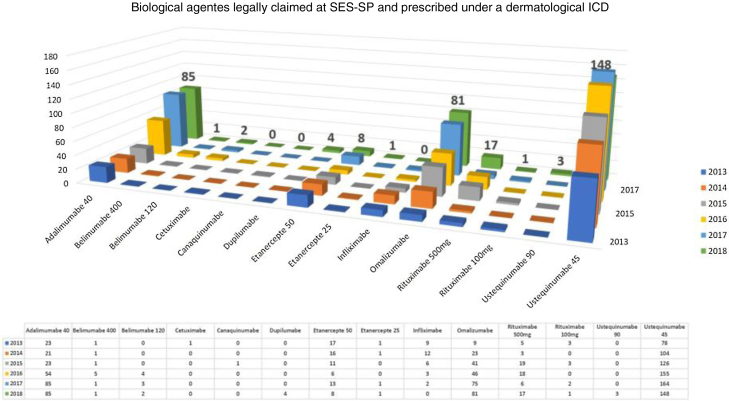
Immunobiologicals provided by the Health Department of the State of São Paulo in the 2013–2018 biennium in obedience to the demands of lawsuits, whose prescribing doctors are listed as dermatologists.

Considering exclusively an analysis of legal claims for immunobiologicals for dermatological reasons at SES-SP in the period of 2013–2018, it can be observed that there is still a predominance of psoriasis as the disease with the most lawsuits in the state (but with a decline in the comparison of years, after the insertion of some immunobiologicals in CEAF). However, the rise of other dermatoses such as hidradenitis suppurative, urticaria, pemphigus, and atopic dermatitis has been observed in the last five years. No cases of legal claims for medication for dermatoses considered to be rare diseases were observed. In 2018, the five most legally claimed dermatological immunobiologicals in the state of São Paulo were ustekinumab, adalimumab, omalizumab, etanercept, and rituximab (Fig. 2).^30^

According to the study carried out in 2013 on new drugs registered in Brazil from the perspective of SUS, eight drugs were registered for five dermatoses (representing 1.3% of all new pharmacological records), and three drugs were indicated for psoriasis. For dermatoses considered by the World Health Organization (WHO) as neglected, such as leprosy and chromoblastomycosis, no medications were incorporated in the records.^21^

Among the 172 drugs included in CEAF until the last update in 2020, Table 2 presents the drugs included for each dermatological ICD.^31^

**Table 2:** List of CEAF drugs per dermatological disease.

Medicine authorized by CEAF	Dermatological disease
Hydroxychloroquine	Discoid lupus erythematosus (DLE), subacute cutaneous lupus erythematosus (SCLE), dermatomyositis
Chloroquine	DLE, SCLE
Cyclophosphamide	DLE, SCLE
Danazol	DLE, SCLE
Injectable human immunoglobulin G	Dermatomyositis
Cyclosporine	DLE, SCLE, dermatomyositis, psoriasis
Azathioprine	DLE, SCLE, dermatomyositis
Methotrexate (MTX)	DLE, SCLE, dermatomyositis, psoriasis
Isotretinoin	Acne
Acitretin	Psoriasis, pityriasis rubra pilar, ichthyosis, hereditary lymphedema, xeroderma pigmentosum, mastocytosis, pigmentary incontinence, ectodermal dysplasia and other congenital skin malformations
Injectable adalimumab	Psoriasis, hidradenitis
Injectable ustekinumab	Psoriasis
Injectable secukinumab	Psoriasis
Injectable etanercept	Psoriasis
Topical calcipotriol	Psoriasis
Clobetasol ointment and hair solution	Psoriasis

The small participation of dermatology within CEAF is notable, which may be the result of the small awareness of managers in relation to dermatological diseases and/or a favoring of diseases considered more serious, with a greater impact on morbidity and mortality rates.

In addition to this issue, it is observed that some drugs of dermatological interest are only considered for other diseases. Data from SES-SP point to rivaroxaban, a new oral anticoagulant used to prevent venous thromboembolism, as the main cause of legal demands. This medication can also be used to prevent and treat thrombotic vasculopathies in dermatology.^27^, ^32^

The term ‘off-label use’ involves the prescription of drugs for non-approved indications, in inappropriate dosages (in relation to those mentioned in the package leaflet), or though unusual administration routes. In dermatology, the use of off-label drugs is very frequent, due to the high cost of clinical studies, the long delay for approval by regulatory agencies, and the difficult clinical management. This way of using medicines is a matter of debate in the medical community, because while it helps patients to control their illnesses, it puts them at risk for certain unwanted and potentially fatal pharmacological effects.

In clinical practice, the multiple interfaces of dermatological disorders with other specialties are noteworthy, as is the possibility of using drugs already used for non-dermatological diseases in skin disorders. Good examples already established worldwide include the use of rituximab (authorized for the treatment of lymphoproliferative diseases) in the control of pemphigus vulgaris and bullous pemphigoid, as well as the use of thalidomide (authorized in SUS for use in case of lupus erythematosus and type-2 leprosy reactions) in cases of recurrent prurigo nodularis.^33^, ^34^

Based on all these difficulties to legitimize the use of certain medications in dermatology, the partnership of academic institutions, the public health system, and the pharmaceutical industry, which is already a reality worldwide, becomes increasingly necessary, aiming to develop research, conduct fast therapeutic trials, provide theoretical and practical assistance for the CONITEC inclusions, improve dermatological education in academic centers, and develop the pharmaceutical market. Brazil has made recent changes to its science, technology, and innovation policies, among which the creation of Sectorial Funds, the Innovation Law, the so-called “Good Law” (Law 11.196/05, on tax exemption), the regulation of the National Fund for Scientific and Technological Development, and the creation of the Pro-Pharma I and II programs of the National Bank for Economic and Social Development are noteworthy.^20^

In the world ranking of clinical research, Brazil occupies only the 24^th^ position, with 2.1% of studies carried out in the world. Sanitary and ethical obstacles to research procedures are the main reasons for this indicator, hindering national pharmaceutical innovation.^35^

### Strategies, mechanisms, and instruments that minimize the legal claims process

As a competency of public managers, it is necessary to develop integrated policies in favor of access and innovation. Therefore, the strategies and measures in Table 3 are suggested.^5^, ^16^, ^20^, ^36^, ^37^, ^38^

**Table 3:** Measures and strategies suggested to minimize legal claims for access to medicines and health supplies.

Define investment priorities: in view of the greater likelihood of patient rehabilitation, severity of the disease, repercussions on society, and pharmacological cost-benefit.
Promote mechanisms to enable the financing and transfer of technology to public health institutions.
Stimulate free competition in the medicine market, as well as optimize sectors within the departments that deal with negotiation, purchase, and logistics.
Establish subsidies for the improvement of institutions and tools such as: the Technical Study Group on Medicines (legitimized by SMS Resolution No. 1,139) NAT and e-NATJUS (digital platform maintained by the CNJ), which assist judges in their decisions.
Investing in pre-judicial mediation systems: avoiding the indiscriminate opening of lawsuits and reversing the concession automatism (Example: extraordinary resources 566471 and 657718, reported by Justice Marco Aurélio, of the STF). Measures such as those carried out by SES-SP at the São Paulo Court of Justice, creating the Acessa SUS project, should be encouraged (in 48,000 cases, 74% of the claims were solved in 2018).
To write and improve RENAME's editions to make them more comprehensible to the responsible judges.
Standardize systems for monitoring legal decisions (such as S-Codes): better tracking and follow-up of deliberations at national level.
Develop continuing medical education programs based on the rational use of CEAF medications and compliance with the PCDT.
Encourage the opening of administrative processes (example: Resolution SS-54, of 11/05/2012 SES-SP) and require medical examination reports for patients who request CEAF drugs, confirming data on previous treatments, complementary exams, and clinical characteristics.

## Financial and administrative aspects

The growing increase in health costs was mainly due to the exponential increase in the use of expensive medicines and their incorporation into health systems.^39^ Economic assessments are based on the opportunity cost, *i.e*., the application of resources in certain technologies implies not providing others. Thus, attention should be paid to therapies with high added cost.^40^

Property law (patents), in addition to changes in the age pyramid and population growth, contribute to rising annual drug costs. In 2018, the MH had R$ 26.38 billion in general expenses; medications accounted for 64% of the total (approximately R$ 16.8 billion), an increase of 41.3% in comparison with the previous year. However, the amount allocated to pharmaceutical assistance was reduced by 4%, with emphasis on the largest reduction (23%) for the specialized component.^35^

Chronologically, the national figures, for 2003 showed the expenses for CEAF were approximately R$ 1.05 billion and R$ 1.92 billion in 2005; after 11 years, they reached R$ 6.6 billion in 2016. Until 2004, dermatology was responsible for only 5.10% of the drugs distributed in this component, with acne vulgaris being the main representative.^41^

The evaluation carried out for the purchase of immunobiologicals used in the control of psoriasis demonstrates the concern in comparing the prices practised internationally and in Brazil. In the general context (evaluating exchange rate variations and tax burdens), Brazil negotiated and purchased the drugs (adalimumab, etanercept, and infliximab) below the international prices observed (unit costs, respectively: R$ 508.61, R$ 291.20, and R$ 901.95). Only secukinumab and ustekinumab were not purchased at the best price in the comparison of purchases; the best acquisitions were made by Colombia (R$ 1,258.61 *vs*. R$ 2,513.90 for Brazil) and South Africa (R$ 6,096.93 *vs.* R$ 9,733.86 for Brazil), respectively. In the same study, the cost until clinical response of non-biological psoriasis drugs (methotrexate, cyclosporine, and acitretin) was analyzed and the extremely low cost compared to immunobiologicals is noted, which again refers to the value of the criteria for indication and medical awareness in terms of prescription.^12^

Considering the incorporation of adalimumab and secukinumab as the first and second lines for psoriasis treatment, respectively, the estimated cost for the first year of approximately R$ 15.6 million is projected according to budgetary impact assessments. After five years of incorporation, the estimated impact would be around R$ 956 million.^12^

In terms of immunobiologicals, due to certain delays in the inclusion/purchase of CEAF drugs, some companies provide the start of treatment until release by the public sector, helping to assist economically underprivileged patients and in turn contributing to a further increase in legal claims for medications.

## Clinical aspects and monitoring of the technological horizon

### Innovations in hidradenitis suppurativa

In July 2018, CONITEC published an opinion in favor of incorporating adalimumab for cases of moderate to severe active hidradenitis. The calculated incremental budget impact was approximately R$ 188 million over five years. In turn, controlling the disease would impact emergency care provided to these patients, reducing costs and hospitalization rates.^42^ Currently, the drug is already available for use, incorporated into the PCDT of the disease.

Some new therapies are being used in studies, such as: anakinra (anti-IL-1), bermekimab (anti IL-1alpha), canakinumab (anti-IL1), and secukinumab (anti IL-17). These can contribute to the blockade of other immunological pathways responsible for the heterogeneity of clinical presentations.^43^

### Treatment of pemphigus and pemphigoid

There are several forms of drug treatment. In the past, oral corticosteroid therapy in high doses was the only way to control the disease; however, many patients ended up suffering the consequences of prolonged use. With the advancement of scientific knowledge, certain medications such as azathioprine, dapsone, methotrexate, and pentoxifylline have come to be used as low-cost, steroid-sparing drugs.^35^

Currently, rituximab (a monoclonal antibody against type B lymphocytes, anti-CD20, used primarily in the treatment of lymphomas) is gaining ground in therapeutic discussions. When compared with conventional therapies, rituximab shows low complication rates and reduced hospitalization rates, justifying the reduction in the annual global cost per patient. These drugs may even be used concurrently, with good safety and efficacy rates.^35^

### Field cancerization treatment

In addition to guidance on photoprotection, several treatments can be performed, from topical treatments (photodynamic therapy, imiquimod, 5-fluoracil) to procedures (curettage, surgery, laser, and cryotherapy), which can be performed alone or in combination. For renal transplant patients, the use of oral acitretin is indicated due to its immunomodulatory power acting in scamous cell carcinoma chemoprophylaxis.^44^

In a study of pharmacoeconomics in the American healthcare scenario, photodynamic therapy was the treatment with the best cost-benefit, best aesthetic result, and best tolerance on the part of the patient. Its advantage is the possibility of being performed under medical supervision and even with the use of sunlight (daylight protocol). 5-fluoracil also showed good cost-effectiveness.^44^

### Clinical management of chronic spontaneous urticaria

Spontaneous chronic urticaria is a subtype of urticaria that affects about 0.1% to 3% of the entire world population. In American economic statistics, this disease costs the government about USD 9,000 per patient per year and the drugs used to control it can start from USD 50 per month (in the case of antihistamines) up to USD 1,000 per month (per injection of omalizumab).^45^ Another topic to be assessed is the high incidence of socio-emotional and psychiatric disorders, which affect work capacity and impact on the annual global cost per patient (including costs with medicines and emergency assessments, in addition to absences from work).

According to international consensus, treatment generally begins with the use of antihistamines and, if there is no control of symptoms, the drug omalizumab (non-specific anti-IgE immunobiological) is indicated, leaving only cyclosporine as the last therapy for refractory and severe cases.^45^

### Indications for the use of systemic retinoids in dermatology

Drugs derived or structurally similar to vitamin A make up the group of retinoids. In Brazil, only acitretin and isotretinoin are authorized by ANVISA and the indications are described in Table 2.

New indications for the use of systemic retinoids (still as off-label indications) should be evaluated. Currently, diseases such as lichen planus, hidradenitis suppurative, chronic hand eczema, viral warts, non-melanoma skin neoplasms, papular pustular rosacea, Darier's disease, and scalp scarring alopecias can be treated with these drugs.^46^

For economic evaluation terms, the highest price of the aforementioned drugs paid by the government, according to ANVISA (updated 10/1/2019, box of 30 tablets) was: R$ 112.33 for acitretin 10 mg, R$ 279.27 for acitretin 25 mg, R$ 83.17 for isotretinoin 10 mg, and R$ 155.82 for isotretinoin 20 mg.^47^

### Thrombotic vasculopathies and the use of anticoagulants in dermatology

In particular, cutaneous thrombotic vasculopathies arise due to a certain factor involving the formation of vascular occlusions; the main form of treatment is the use of anticoagulants. In terms of cost-effectiveness, in analyses involving other non-dermatological disease, the new direct oral anticoagulants (*e.g*., dabigatran, rivaroxaban, and apixaban) were shown to be more efficient than enoxaparin, under criteria including costs with exams, consultations, medicine cost, and safety profile.^48^

The dermatological indications for oral anticoagulants are livedoid vasculopathy, purpura fulminans, superficial venous thrombosis, antiphospholipid syndrome, Raynaud's phenomenon, calciphylaxis, lichen planus, livedo racemosa, and chronic venous ulcers.^33^

### Challenges in severe cases of atopic dermatitis

When considering that atopic dermatitis can occur at any age and that there are several forms of treatment and prevention, high economic impacts (direct and indirect) are expected for the public and supplementary health systems. New topical drugs are being used to protect the integrity of the skin barrier (*e.g*., phosphodiesterase-4 inhibitors and Janus kinase inhibitors) and certain systemic drugs are emerging on the market for use in patients resistant to conventional therapies (cyclosporine, methotrexate, and mycophenolate mofetil), such as dupilumab (human monoclonal antibody against interleukin IL-4 alpha and IL-3) which, despite the high cost per patient, presents good clinical results.^49^

### Future considerations for the treatment of psoriasis

Faced with a scenario of technological innovations and in the era of immunobiologicals, new therapies are emerging with the aim of blocking different immunological pathways of the disease. The most recent biological products on the market and approved by the Food and Drug Administration (FDA) work to improve the inhibition of interleukins IL-23 (guselkumab, tildrakizumab, and risankizumab) and IL-17 (ixekizumab, brodalimumab).^50^

There is a wide discussion on the issue of the patents of these immunobiologicals and the coming forth of biosimilars. This last class of drugs is intended to decrease the final cost and improve access to treatment. The equivalence between biologicals and biosimilars is a very debated issue in the medical field and criteria are still lacking to ensure the interchangeability of these classes or extrapolation of clinical data (with pharmacovigilance profiles). Thus, the automatic substitution of an immunobiological by its biosimilar, in addition to resulting in interchangeability without a therapeutic purpose (considering a patient who responds well to the immunobiological), may represent a disregard for the autonomy of the prescribing physician.

## Conclusions

In the present Brazilian context of a continental country in full economic development and with different realities in terms of the public health system, planning the organization of access to CEAF appears to be one of the priorities for health managers. From the drug insertion project to its distribution, many financial and product value chain analyses are needed.

Reassessing criteria for obtaining medication at a high cost should be a practice to be disseminated by all national law providers, as well as making decisions based on the current socioeconomic context of the state. States and the PJ must work together to unify indicators and conduct pre-judicial mediation of conflicts, with the ultimate goal of reducing legal claims in health.

Currently, the dermatological practice within SUS still lacks specialized therapeutic medications. Taking stock of the results and the efficacy and safety profiles of new drugs used worldwide in dermatology could be a starting point for Brazilian therapeutic resolutions, with scientific and administrative basis for national health decisions.

## Financial support

None declared.

## Authors’ contributions

The main/corresponding author declares for due purposes, that all co-authors participated in afull form at all stages of the preparation of the scientific article (assembly and data collection; tabulation, analysis, and creation of tables/figures; text writing); text review and adjustment to the magazine's rules).

## Conflicts of interest

None declared.
